# Supplementary material for: Health impact of the COVID-19 in Spanish non-healthcare workers by gender: Use of sickness absence for surveillance
Source: PLoS One. 2024 Oct 9;19(10):e0307224. doi: 10.1371/journal.pone.0307224 (PMC11463834; doi:10.1371/journal.pone.0307224)
Supplement: S1 Table — Spain, February 15th to September 17th, 2020. (DOCX) [file pone.0307224.s001.docx]

**S1 Table . Temporary disability due to COVID-19, per 100,000 workers, by economic activity (excluding the healthcare and social healthcare sectors), by sex. Spain, February 15^th^ to September 17^th^, 2020.**

| **Economic activity division (two-digit numerical codes NACE)** | **Female** | | | | **Male** | | | |
| --- | --- | --- | --- | --- | --- | --- | --- | --- |
|  | **% FW** | **% TD** | **NTD** | **TDREA** | **% MW** | **% TD** | **NTD** | **TDREA** |
| 01. Crop and animal production, hunting and related service activities | 1.29 | 2.09 | 2,338 | **2,598** | 2.34 | 3.08 | 4,005 | **1,830** |
| 02.Forestry and logging | 0.05 | 0.03 | 31 | **961** | 0.23 | 0.24 | 306 | **1,414** |
| 03.Fishing and aquaculture | 0.01 | 0.01 | 11 | **1,217** | 0.02 | 0.13 | 166 | **8,136** |
| 05. Mining of coal and lignite | 0.00 | 0.00 | 0 | **0** | 0.00 | 0.00 | 4 | **3,195** |
| 06.Extraction of crude petroleum and natural gas | 0.00 | 0.00 | 0 | **0** | 0.00 | 0.01 | 7 | **5,220** |
| 07. Mining of metal ores | 0.01 | 0.00 | 0 | **0** | 0.03 | 0.01 | 15 | **537** |
| 08. Other mining and quarrying | 0.02 | 0.02 | 18 | **1,054** | 0.15 | 0.14 | 180 | **1,274** |
| 09. Mining support service activities | 0.01 | 0.01 | 7 | **1,899** | 0.01 | 0.00 | 2 | **232** |
| 10. Manufacture of food products | 2.22 | 3.65 | 4,078 | **2,632** | 2.51 | 5.36 | 6,973 | **2,969** |
| 11. Manufacture of beverages | 0.19 | 0.17 | 192 | **1,459** | 0.37 | 0.44 | 578 | **1,659** |
| 12. Manufacture of tobacco products | 0.01 | 0.00 | 2 | **304** | 0.01 | 0.00 | 5 | **502** |
| 13. Manufacture of textiles | 0.28 | 0.25 | 284 | **1,455** | 0.29 | 0.31 | 407 | **1,498** |
| 14. Manufacture of wearing apparel | 0.49 | 0.28 | 311 | **915** | 0.14 | 0.09 | 114 | **850** |
| 15. Manufacture of leather and related products | 0.29 | 0.12 | 130 | **650** | 0.26 | 0.11 | 147 | **607** |
| 16. Manufacture of wood and of products of wood and cork, except furniture; manufacture of articles of straw and plaiting materials | 0.13 | 0.09 | 102 | **1,144** | 0.57 | 0.47 | 613 | **1,154** |
| 17. Manufacture of paper and paper products | 0.14 | 0.15 | 167 | **1,701** | 0.36 | 0.55 | 713 | **2,133** |
| 18. Printing and reproduction of recorded media | 0.32 | 0.24 | 273 | **1,207** | 0.54 | 0.54 | 706 | **1,402** |
| 19. Manufacture of coke and refined petroleum products | 0.02 | 0.02 | 23 | **1,349** | 0.07 | 0.09 | 119 | **1,746** |
| 20. Manufacture of chemicals and chemical products | 0.43 | 0.46 | 512 | **1,711** | 0.74 | 1.07 | 1,396 | **2,019** |
| 21. Manufacture of basic pharmaceutical products and pharmaceutical preparations | 0.38 | 0.73 | 815 | **3,088** | 0.29 | 0.64 | 826 | **3,086** |
| 22.Manufacture of rubber and plastic products | 0.36 | 0.42 | 469 | **1,891** | 0.74 | 0.94 | 1,226 | **1,777** |
| 23.Manufacture of other non-metallic mineral products | 0.23 | 0.15 | 169 | **1,052** | 0.88 | 0.87 | 1,136 | **1,383** |
| 24. Manufacture of basic metals | 0.13 | 0.11 | 128 | **1,465** | 0.74 | 0.81 | 1,055 | **1,518** |
| 25. Manufacture of fabricated metal products, except machinery and equipment | 0.54 | 0.51 | 568 | **1,500** | 2.39 | 2.66 | 3,455 | **1,547** |
| 26.Manufacture of computer, electronic, and optical products | 0.13 | 0.35 | 390 | **4,459** | 0.22 | 0.51 | 669 | **3,264** |
| 27.Manufacture of electrical equipment | 0.17 | 0.16 | 174 | **1,498** | 0.37 | 0.47 | 606 | **1,743** |
| 28.Manufacture of machinery and equipment n.e.c. | 0.30 | 0.26 | 293 | **1,394** | 1.15 | 1.44 | 1,878 | **1,739** |
| 29.Manufacture of motor vehicles, trailers, and semitrailers | 0.52 | 0.53 | 588 | **1,636** | 1.36 | 1.49 | 1,935 | **1,519** |
| 30.Manufacture of other transport equipment | 0.13 | 0.12 | 133 | **1,430** | 0.51 | 0.54 | 699 | **1,455** |
| 31. Manufacture of furniture | 0.17 | 0.12 | 130 | **1,119** | 0.56 | 0.49 | 637 | **1,209** |
| 32.Other manufacturing | 0.24 | 0.16 | 178 | **1,084** | 0.23 | 0.18 | 235 | **1,099** |
| 33.Repair and installation of machinery and equipment | 0.19 | 0.13 | 149 | **1,140** | 0.93 | 1.06 | 1,373 | **1,571** |
| 35. Electricity, gas, steam and air conditioning supply | 0.12 | 0.07 | 82 | **963** | 0.29 | 0.25 | 325 | **1,187** |
| 36. Water collection, treatment and supply | 0.13 | 0.11 | 121 | **1,301** | 0.37 | 0.35 | 459 | **1,343** |
| 37. Sewerage | 0.01 | 0.01 | 16 | **1,596** | 0.05 | 0.06 | 82 | **1,732** |
| 38. Waste collection, treatment and disposal activities; materials recovery | 0.26 | 0.33 | 374 | **2,084** | 0.81 | 1.15 | 1,492 | **1,961** |
| 39. Remediation activities and other waste management services | 0.01 | 0.01 | 8 | **1,611** | 0.02 | 0.02 | 21 | **1,353** |
| 41.Construction of buildings | 0.88 | 0.52 | 587 | **959** | 4.34 | 3.60 | 4,685 | **1,154** |
| 42.Civil engineering | 0.08 | 0.05 | 57 | **1,025** | 0.52 | 0.51 | 666 | **1,373** |
| 43.Specialized construction activities | 1.01 | 0.59 | 656 | **937** | 7.17 | 6.58 | 8,557 | **1,276** |
| 45. Wholesale and retail trade and repair of motor vehicles and motorcycles | 0.78 | 0.44 | 489 | **903** | 3.06 | 2.12 | 2,757 | **964** |
| 46. Wholesale trade, except of motor vehicles and motorcycles | 5.20 | 4.79 | 5,355 | **1,479** | 6.81 | 6.29 | 8,180 | **1,284** |
| 47.Retail trade, except motor vehicles and motorcycles | 16.32 | 15.67 | 17,525 | **1,541** | 7.82 | 6.97 | 9,062 | **1,239** |
| 49.Land transport and transport via pipelines | 1.09 | 0.95 | 1,060 | **1,400** | 5.71 | 5.04 | 6,551 | **1,226** |
| 50. Water transport | 0.01 | 0.02 | 25 | **5,718** | 0.01 | 0.08 | 101 | **15,373** |
| 51.Air transport | 0.23 | 0.28 | 313 | **1,965** | 0.24 | 0.43 | 558 | **2,499** |
| 52. Warehousing and support activities for transportation | 0.89 | 1.83 | 2,048 | **3,291** | 1.48 | 2.57 | 3,344 | **2,411** |
| 53.Postal and courier activities | 0.51 | 0.69 | 773 | **2,187** | 0.54 | 0.80 | 1,037 | **2,056** |
| 55.Accommodation | 2.12 | 1.40 | 1,565 | **1,061** | 1.33 | 0.95 | 1,231 | **991** |
| 56.Food and beverage service activities | 9.65 | 6.94 | 7,762 | **1,154** | 6.61 | 4.88 | 6,350 | **1,026** |
| 58.Publishing activities | 0.32 | 0.19 | 209 | **951** | 0.30 | 0.18 | 231 | **830** |
| 59. Motion picture, video and television programme production, sound recording and music publishing activities | 0.27 | 0.22 | 242 | **1,300** | 0.29 | 0.23 | 305 | **1,108** |
| 60.Programming and broadcasting activities | 0.16 | 0.19 | 208 | **1,882** | 0.16 | 0.23 | 296 | **1,991** |
| 61.Telecommunications | 0.36 | 0.27 | 305 | **1,202** | 0.54 | 0.46 | 594 | **1,184** |
| 62. Computer programming, consultancy and related activities | 1.47 | 0.77 | 863 | **843** | 2.57 | 1.51 | 1,967 | **819** |
| 63.Information services activities | 0.23 | 0.19 | 214 | **1,331** | 0.21 | 0.17 | 218 | **1,091** |
| 64.Financial services, except insurance and pension funding | 1.52 | 1.59 | 1,780 | **1,680** | 1.10 | 1.13 | 1,467 | **1,431** |
| 65.Insurance, reinsurance, and pension funding, except compulsory social security | 0.47 | 0.45 | 500 | **1,519** | 0.29 | 0.23 | 299 | **1,088** |
| 66.Activities auxiliary to financial services and insurance activities | 0.87 | 0.54 | 605 | **996** | 0.55 | 0.25 | 324 | **630** |
| 68.Real estate activities | 1.16 | 0.61 | 678 | **842** | 0.71 | 0.42 | 544 | **817** |
| 69.Legal and accounting activities | 2.65 | 1.82 | 2,032 | **1,100** | 1.34 | 0.75 | 969 | **774** |
| 70.Activities of head offices; management consultancy activities | 0.92 | 0.63 | 701 | **1,091** | 0.62 | 0.39 | 511 | **881** |
| 71. Architectural and engineering activities; technical testing and analysis | 1.22 | 0.70 | 785 | **925** | 1.84 | 1.16 | 1,504 | **872** |
| 72. Scientific research and development | 0.70 | 0.51 | 574 | **1,173** | 0.47 | 0.35 | 455 | **1,044** |
| 73.Advertising and market research | 0.86 | 0.42 | 473 | **789** | 0.59 | 0.27 | 352 | **637** |
| 74.Other professional, scientific, and technical activities | 1.03 | 0.64 | 720 | **1,661** | 0.73 | 0.46 | 598 | **1,400** |
| 75.Veterinary activities | 0.26 | 0.31 | 342 | **1,901** | 0.10 | 0.06 | 73 | **809** |
| 77.Rental and leasing activities | 0.47 | 0.33 | 374 | **1,141** | 0.64 | 0.48 | 621 | **1,029** |
| 78.Employment activities | 1.25 | 2.43 | 2,722 | **3,128** | 1.24 | 2.96 | 3,855 | **3,311** |
| 79. Travel agency, tour operator and other reservation service and related activities | 0.60 | 0.26 | 293 | **707** | 0.24 | 0.09 | 120 | **533** |
| 80.Security and investigation activities | 0.35 | 0.63 | 705 | **2,916** | 1.20 | 1.87 | 2,426 | **2,168** |
| 81. Services to buildings and landscape activities | 5.76 | 9.62 | 10,757 | **2,682** | 2.19 | 3.14 | 4,085 | **1,997** |
| 82. Office administrative, office support and other business support activities | 2.56 | 3.72 | 4,157 | **2,329** | 1.67 | 2.43 | 3,156 | **2,025** |
| 84.Public administration and defence; compulsory social security | 8.31 | 9.18 | 10,267 | **1,774** | 5.72 | 7.76 | 10,092 | **1,887** |
| 85.Education | 10.38 | 4.63 | 5,184 | **717** | 3.99 | 2.19 | 2,849 | **764** |
| 90.Creative, arts and entertainment activities | 0.38 | 0.12 | 130 | **487** | 0.40 | 0.12 | 158 | **419** |
| 91.Library, archives, museums and other cultural activities | 0.14 | 0.10 | 116 | **1,197** | 0.08 | 0.06 | 76 | **1,007** |
| 92.Gambling and betting activities | 0.26 | 0.17 | 185 | **1,033** | 0.21 | 0.13 | 165 | **845** |
| 93. Sports activities and amusement and recreation activities | 1.32 | 0.87 | 971 | **1,053** | 1.42 | 1.06 | 1,381 | **1,037** |
| 94. Activities of membership organisations | 1.25 | 1.22 | 1,370 | **1,576** | 0.60 | 0.46 | 596 | **1,070** |
| 95.Repair of computers and household goods | 0.25 | 0.07 | 81 | **471** | 0.53 | 0.30 | 389 | **792** |
| 96.Other personal service activities | 3.42 | 1.63 | 1,823 | **766** | 0.98 | 0.67 | 868 | **947** |
| 97.Activities of households as employers of domestic personnel | 0.19 | 8.94 | 10,004 | **76,663** | 0.31 | 0.66 | 863 | **2,990** |
| **Global** | 100.00 | 100.00 | 111,849 | **1,605** | 100.00 | 100.00 | 130,051 | **1,390** |

NACE: Statistical Classification of Economic Activities in the European Community// FW: Female workers// MW: Male Workers// TD: Temporary disability //NTD: Number of temporary disabilities // TDREA: Temporary disability rate by economic activity
