# Supplementary material for: Health impact of the COVID-19 in Spanish non-healthcare workers by gender: Use of sickness absence for surveillance
Source: PLoS One. 2024 Oct 9;19(10):e0307224. doi: 10.1371/journal.pone.0307224 (PMC11463834; doi:10.1371/journal.pone.0307224)
Supplement: S2 Table — Spain, February 15th to September 17th, 2020. (DOCX) [file pone.0307224.s002.docx]

**S2 Table. Temporary disability due to COVID-19, per 100,000 workers, by occupation (excluding the health and social care personnel), by sex. Spain, February 15^th^ to September 17^th^, 2020.**

| **Occupation (two-digit numerical codes ISCO)** | **Female** | | | | **Male** | | | |
| --- | --- | --- | --- | --- | --- | --- | --- | --- |
|  | **% FW** | **% TD** | **NTD** | **TDRO** | **% MW** | **% TD** | **NTD** | **TDRO** |
| 11. Chief Executives, Senior Officials and Legislators | 0.26 | 0.26 | 231 | **1,134** | 0.32 | 0.31 | 374 | **1,146** |
| 12. Administrative and Commercial Managers | 1.01 | 0.90 | 787 | **1,006** | 1.02 | 1.36 | 1,627 | **1,560** |
| 13. Production and Specialized Services Managers | 0.96 | 0.56 | 492 | **656** | 1.49 | 1.15 | 1,378 | **904** |
| 14. Hospitality, Retail and Other Services Managers | 1.18 | 0.84 | 735 | **797** | 1.90 | 1.46 | 1,752 | **902** |
| 21. Science and Engineering Professionals | 2.24 | 1.40 | 1,219 | **699** | 3.86 | 2.96 | 3,544 | **897** |
| 22. Teaching Professionals | 9.78 | 3.06 | 2,674 | **351** | 3.51 | 1.15 | 1,375 | **383** |
| 24. Business and Administration Professionals | 3.34 | 1.72 | 1,500 | **577** | 2.09 | 1.31 | 1,577 | **739** |
| 25. Information and Communications Technology Professionals | 0.56 | 0.68 | 594 | **1,351** | 1.61 | 1.17 | 1,405 | **853** |
| 26. Legal, Social and Cultural Professionals | 4.46 | 2.35 | 2,050 | **591** | 2.55 | 1.20 | 1,440 | **551** |
| 31. Science and Engineering Associate Professionals | 1.23 | 1.32 | 1,150 | **1,201** | 3.59 | 4.27 | 5,116 | **1,394** |
| 32. Health Associate Professionals | 1.13 | 1.08 | 939 | **1,064** | 0.39 | 0.48 | 575 | **1,443** |
| 33. Business and Administration Associate Professionals | 5.88 | 4.99 | 4,355 | **951** | 5.23 | 3.98 | 4,774 | **893** |
| 34. Legal, Social, Cultural and Related Associate Professionals | 1.82 | 1.39 | 1,215 | **856** | 1.66 | 0.91 | 1,089 | **641** |
| 35. Information and Communications Technicians | 0.63 | 0.46 | 400 | **818** | 2.26 | 2.04 | 2,452 | **1,063** |
| 41. General and Keyboard Clerks | 4.80 | 4.72 | 4,118 | **1,101** | 1.38 | 1.60 | 1,923 | **1,366** |
| 42. Customer Services Clerks | 8.72 | 11.82 | 10,325 | **1,522** | 2.53 | 3.62 | 4,340 | **1,680** |
| 43. Numerical and Material Recording Clerks | 3.62 | 2.42 | 2,112 | **750** | 2.31 | 2.09 | 2,506 | **1,063** |
| 44. Other Clerical Support Workers | 0.51 | 0.65 | 567 | **1,419** | 0.35 | 0.44 | 522 | **1,457** |
| 51. Personal Service Workers | 10.14 | 12.84 | 11,214 | **1,421** | 7.10 | 8.18 | 9,811 | **1,351** |
| 52. Sales Workers | 12.80 | 4.95 | 4,324 | **434** | 5.33 | 2.25 | 2,699 | **495** |
| 54. Protective Services Workers | 0.69 | 1.65 | 1,439 | **2,662** | 3.64 | 5.58 | 6,695 | **1,797** |
| 61. Market-oriented Skilled Agricultural Workers | 0.95 | 0.72 | 630 | **855** | 3.04 | 1.49 | 1,791 | **576** |
| 62. Market-Oriented Skilled Forestry, Fishery and Hunting Workers | 0.04 | 0.03 | 23 | **743** | 0.33 | 0.13 | 153 | **457** |
| 71. Building and Related Trades Workers (excluding Electricians) | 0.19 | 0.46 | 400 | **2,735** | 8.37 | 8.23 | 9,866 | **1,153** |
| 72. Metal, Machinery and Related Trades Workers | 0.16 | 0.24 | 210 | **1,644** | 5.44 | 4.54 | 5,443 | **979** |
| 73. Handicraft and Printing Workers | 0.26 | 0.21 | 186 | **904** | 0.60 | 0.52 | 626 | **1,017** |
| 74. Electrical and Electronics Trades Workers | 0.07 | 0.05 | 40 | **698** | 3.16 | 2.15 | 2,584 | **798** |
| 75. Food Processing, Woodworking, Garment and Other Craft and Related Trades Workers | 1.42 | 4.79 | 4,180 | **3,788** | 2.04 | 6.27 | 7,519 | **3,605** |
| 81. Stationary Plant and Machine Operators | 1.69 | 1.89 | 1,652 | **1257** | 2.96 | 2.73 | 3,277 | **1,083** |
| 82. Assemblers | 0.45 | 0.15 | 131 | **373** | 1.00 | 1.04 | 1,249 | **1,224** |
| 83. Drivers and Mobile Plant Operators | 0.47 | 0.59 | 514 | **1,404** | 8.96 | 8.37 | 10,043 | **1,097** |
| 91. Cleaners and Helpers | 12.99 | 20.44 | 17,853 | **1,766** | 1.10 | 1.95 | 2,337 | **2,073** |
| 92. Agricultural, Forestry and Fishery Labourers | 1.06 | 2.94 | 2,570 | **3,101** | 2.69 | 3.25 | 3,893 | **1,415** |
| 93. Construction and mining labourers | 2.48 | 3.93 | 3,432 | **1,776** | 4.60 | 7.37 | 8,836 | **1,876** |
| 94.Food preparation assistants | 1.45 | 1.58 | 1,377 | **1,224** | 0.49 | 0.69 | 823 | **1,635** |
| 95. Street and Related Sales and Service Workers  96. Refuse Workers and Other Elementary Workers | 0.52 | 1.94 | 1,698 | **4,188** | 1.11 | 3.76 | 4,513 | **3,971** |
| **Global** | **100.00** | **100.00** | **87,336** | **1,122** | **100.00** | **100.00** | **119,927** | **1,146** |

ISCO: International Standard Classification of Occupations// FW: Female workers// MW: Male Workers// TD: Temporary disability //NTD: Number of temporary disabilities // TDRO: Temporary disability rate by occupation
