# Supplementary material for: Health impact of the COVID-19 in Spanish non-healthcare workers by gender: Use of sickness absence for surveillance
Source: PLoS One. 2024 Oct 9;19(10):e0307224. doi: 10.1371/journal.pone.0307224 (PMC11463834; doi:10.1371/journal.pone.0307224)
Supplement: Attachment — Submitted filename: Response to Reviewers.docx [file pone.0307224.s003.docx]

**Revisión EDITOR
Journal requirements:**

When submitting your revision, we need you to address these additional requirements.

**Comment 1:**

1. Please ensure that your manuscript meets PLOS ONE's style requirements, including those for file naming. The PLOS ONE style templates can be found at
   https://journals.plos.org/plosone/s/file?id=wjVg/PLOSOne_formatting_sample_main_body.pdf and
   <https://journals.plos.org/plosone/s/file?id=ba62/PLOSOne_formatting_sample_title_authors_affiliations.pdf>

**Reply:** We reviewed the suggested requirements and we believe that the new document meets the journal criteria.

**Comment 2:**

2. Please amend your manuscript to include your abstract after the title page.

**Reply:** We have included the abstract after the title page.

**Comment 3:**

3. In the online submission form you indicate that your data is not available for proprietary reasons and have provided a contact point for accessing this data. Please note that your current contact point is a co-author on this manuscript. According to our Data Policy, the contact point must not be an author on the manuscript and must be an institutional contact, ideally not an individual. Please revise your data statement to a non-author institutional point of contact, such as a data access or ethics committee, and send this to us via return email. Please also include contact information for the third party organization, and please include the full citation of where the data can be found.

**Reply:** Thank you for this comment. A statement will be included in the corresponding website section regarding data availability.

“*The data underlying the results presented in the study are available, after request, from the social security website:*

*https://sede.seg-social.gob.es/wps/portal/sede/sede/Ciudadanos/2022estadisticas/090316_c_ai?*

*changeLanguage=en*”

**Comment 4:**

4. Please include captions for your Supporting Information files at the end of your manuscript, and update any in-text citations to match accordingly. Please see our Supporting Information guidelines for more information: http://journals.plos.org/plosone/s/supporting-information.

**Reply:** We have changed in the text the captions for supplementary material to S1 Table and S2 Table.

We have included supporting information at the end of manuscript

Reviewers' comments:

Reviewer's Responses to Questions

**Comments to the Author**

1. Is the manuscript technically sound, and do the data support the conclusions?

The manuscript must describe a technically sound piece of scientific research with data that supports the conclusions. Experiments must have been conducted rigorously, with appropriate controls, replication, and sample sizes. The conclusions must be drawn appropriately based on the data presented.

Reviewer #1: Yes

Reviewer #2: Partly

Reviewer #3: Partly

2. Has the statistical analysis been performed appropriately and rigorously?

Reviewer #1: Yes

Reviewer #2: No

Reviewer #3: No

3. Have the authors made all data underlying the findings in their manuscript fully available?

The [PLOS Data policy](http://www.plosone.org/static/policies.action#sharing) requires authors to make all data underlying the findings described in their manuscript fully available without restriction, with rare exception (please refer to the Data Availability Statement in the manuscript PDF file). The data should be provided as part of the manuscript or its supporting information, or deposited to a public repository. For example, in addition to summary statistics, the data points behind means, medians and variance measures should be available. If there are restrictions on publicly sharing data—e.g. participant privacy or use of data from a third party—those must be specified.

Reviewer #1: Yes

Reviewer #2: Yes

Reviewer #3: No

4. Is the manuscript presented in an intelligible fashion and written in standard English?

PLOS ONE does not copyedit accepted manuscripts, so the language in submitted articles must be clear, correct, and unambiguous. Any typographical or grammatical errors should be corrected at revision, so please note any specific errors here.

Reviewer #1: Yes

Reviewer #2: No

Reviewer #3: Yes

5. Review Comments to the Author

Please use the space provided to explain your answers to the questions above. You may also include additional comments for the author, including concerns about dual publication, research ethics, or publication ethics. (Please upload your review as an attachment if it exceeds 20,000 characters)

**Reviewer #1: Peer-Review for PLOS ONE**

Date of the review
• 31.01.2024

Title of the manuscript
• "Health impact of the COVID-19 in Spanish workers by gender: use of sickness absence for surveillance.”

Manuscript Identifier:
• PONE-D-24-01071
• First draft

Authors of the draft:
• Not blinded to the reviewer

Type of the manuscript
• primary study

Reviewer's report

Thank you for the opportunity to review the manuscript.

The aim of the study is describing the impact of the pandemic on the Spanish non-healthcare workers measured through health-related workplace absences based on disability data provided by the National Social Security. Temporary disability rates due to Covid-19 by economic activity and be occupation and ratios of the distribution of temporary disability due to quarantine and to Covid-19-illness are used to describe risks.

Scientific background, the aim of the study, hypothesis, statistical methods, results and the discussion are understandable, well and compact written and provide an impressive and good scientific view to the topic.

The reviewers have no additional considerations, recommendations or comments on the title, abstract, keywords, introduction, objective, methods, tables and the figure, the discussion and other aspects (references, acknowledgements etc.). There are no points left. The reviewer recommends the publication of the manuscript in PLOS ONE.

**Reply:**

Thank you very much for your comments.

**Reviewer #2:**

 In this study the authors have investigated the share of temporary disability among non-healthcare working population in the two first waves of the pandemic in Spain. Temporary disability is defined as persons either in quarantine or sick due to covid-19 infection. The authors have investigated this based on gender, age, and occupation.

After carefully reading the paper, the general feeling is that the paper seems a bit unfished. There are several spelling errors, repetitive sentences, and the paper is missing a “story”. I therefore do not believe the paper is ready to be published yet.

**Comment 1:**

Introduction:
1) The introduction is working fine. However, I miss a specific knowledge gap this paper is trying to fill. Why is this important and needed? Why is there a need to look at both economic activity and occupation? What new and needed information does this bring?

**Reply:** Thank you for this comment and the relevant questions. We have adapted a paragraph with the suggested information in the introduction:

*“In previous studies, different papers have described the impact of COVID-19 in terms of TD in the healthcare and sociosanitary population (7-12). However, it is important to highlight that the beginning of the second wave in Spain and later in Europe had its origin in Agriculture seasonal workers (6). This shows the relevance of monitoring the work settings and the working and living conditions of workers to better understand the COVID-19 transmission mechanisms. Notwithstanding this, there are very few studies describing the magnitude of the impact on the non-healthcare working population (6, 13, 14). Consequently, our current knowledge of the COVID-19 impact in these workers is still incomplete.”*

**Comment 2:**

Methodology:
1) In the methodology section the "economic activity analysis" and the "occupation analysis" are too scarcely described. It is unclear to me why there is a need for both these analyses, and exactly what the differences are.

**Reply:**

Thank you for your comments, we have reorganised the second and third paragraph of the “Methodology” section in order to make it easier to understand:

*“We use the European Classification of Economic Activities (NACE) (13) for the analysis by economic activity and the International Classification of Occupations (ISCO) (14) for the analysis by occupation. These classifications provide good indicators of the working conditions of workers in a complementary way.*

*For the economic activity analysis, healthcare and sociosanitary activities were excluded (Q86, Q87, and Q88 codes from the NACE. For the occupation analysis, healthcare and personal care professionals were also excluded (codes 21, 56 and 57 of the ISCO).”*

**Comment 3:**

2) Moreover, there is not enough information on what the National Institute of Social Security contains of information: Gender? Age? Occupation? Socio-economic status?

**Reply:**

The social security database contains information structured in multiple variables. For achieving the objectives of this study, we requested only several needed variables: age, gender, NACE code, ISCO code and ICD code (to determine the sickness absence nature). We have included the following information in the first paragraph of the methodology, in order to clarify this point:

*“The cases under study were the processes of temporary disabillity due to COVID-19 (ICD-10 ES codes: B34.2, B97.21, and U07.1) and quarantine due to close contact with a COVID-19 case (ICD-10 ES code: Z20.828) from February 15 to September 17, 2020, provided by the National Institute of Social Security (INSS), by age, gender, economic activity and occupation.”*

**Comment 4:**

3) The National Institute of Social Security is shortened to INSS at the start of the section, but throughout the rest of the paper the authors refer to something they call “social security”. Is this the same? If so, why not use INSS, or why not write National Institute of Social Security (hereafter referred to as “social security”)?

**Reply:**

Thank you for this coment. We checked the text and used the INSS term when we refer to the public institution and “social security” when we mean the service that the institution offers.

**Comment 5:**

4) Finally, the bullet points make the paper look not ready for publication. This should rather be written in clear text, e.g., like: “The following rates of COVID-19 temporary disability processes were obtained as rates by (1) economic activity, and (2) occupation. Economic activity was defined as …”

**Reply:** We modified the text in order to eliminate the bullet points:

*“The following rates of COVID-19 temporary disability processes were obtained as rates by (1) economic activity, and (2) occupation.*

*COVID-19 Temporary disabillity Rate by Economic Activity (TDREA) was defined as: Number of temporary disabillity processes (NTD) in a NACE code per 100,000 active affiliates to Social Security in that NACE code.*

$$TDREA=\frac{NTD by NACE code*100,000}{Number of active affiliates to Social Security by NACE code}$$

*COVID-19 Temporary disabillity Rate by Occupation (TDRO) was defined as: Number of temporary disabillity processes in an ISCO code per 100,000 employed individuals in that ISCO code.*

$$TDRO=\frac{NTD By ISCO code*100,000}{Number of employed individuals by ISCO code}$$

**“**

**Comment 6:**

**Results:**
1) The start of the results section with descriptive information works well.

2) However, the rest of the section feels too much of a series of listings, which can disrupt the flow of the text. I would rather suggest trying to get a better structure by grouping together similar occupations, or explicitly state the structure of the results. “Highest TD”, “Highest illness”, etc.

Reply: We believe that you are right to look for better groupings and that is what we have tried to do throughout the writing of the article, however, there is a lot of heterogeneity in the results so that the information we present is already a summary of a large number of occupational sectors. The occupations presented already constitute a grouping according to occupation categories.

**Comment 7:**

3) In Table 1 it is hard to understand what are decimals and what are not. I would suggest not using punctuation mark as a thousand separator, but rather using a space: 42 000 instead of 42.000.

**Reply:**

Thank you for this observation. We have standardised the criteria according to the magazine's suggestions.

**Comment 8:**

**Discussion:**
1) I like the comparison to other studies and the possible explanations as to why some numbers are high. This enhances the quality of the paper.
2) “This analysis of TD reveals differences by gender, economic activity and occupation, allowing us to understand the work settings that have posed a higher risk of exposure and, indirectly, identify areas of COVID-19 transmission not adequately captured by other source of information (4).» Here it is pivotal to note what differences. Not just that the paper reveals differences.

**Reply:** Thank you for this coment. We have added additional information in order to point out these important differences in the first paragraph of the Discusion:

*“The results revealed the high impact in terms of sick leave associated with COVID-19 in Spanish workers and important differences by gender, economic activity and occupation that have characterized this impact. The TD rate was 26 and 9.5 times higher for women than for men in the Activities of households as employers of domestic personnel and in the Temporary employment agency activities, respectively. Waste Collection, Activities of call centers, and Processing and preserving of meat products, were also sectors with high risk of exposure for both genders. Taking into account the workers occupation, the less qualified presented the highest rates.”*

Afterwards, we further explore and discuss these differences in the rest of the discution section.

**Comment 9:**

3) On the other hand, I am not sure that the paper allows the authors to state this, as they have not adjusted for things like e.g., socio-economy. To illustrate this, in Norway during the first wave of the pandemic dentists were disproportionately affected on infections. People believed that this was due to the fact that they were so close to a patient’s mouth and nose and with a high risk of being infected. However, as more details emerged, it became apparent that dentists, often belonging to higher socio-economic brackets, had participated in skiing holidays in the Austrian Alps, which marked the onset of one of Europe's first super-spreader events.

**Reply:** We agree with your statement and are aware that the events you mention are real and may influence the analysis of the results. We have included a comment within the limitations that relates to your comments:

*“Associating infections with places and jobs, that is, with certain working conditions, does not incorporate living conditions, commutes to workplaces, and other socioeconomic and environmental determinants, which are relevant to characterize the pandemic and were relevant in outbreaks of slaughterhouses and seasonal workers, for example*.”

**Comment 10:**

4) “We also show that TD is a valuable supplementary source of information for analyzing the impact of COVID-19 in work settings different from health care sector” How?

Actually, the utility of the TD as complememntary source of information for the traditional surveillance system, has been described in another research. In order to clarify this we have modified the phrase you mention, with the corresponding bibliography:

*“TD already was prooven as a useful tool to complement the traditional surveillance system (7,19)”*

**Comment 11:**

5) The second paragraph feels just like a repetition of the results.

**Reply:** We have adapted the paragraph to take account your suggestion:

*“The average age of all TD cases was 42 years, both in men and women, similar to findings in other studies conducted on the working population (20). The highest number of COVID-19 TD cases occurs in middle-aged adults, however, the highest incidence rates of TD due to illness were recorded in younger age groups. The higher impact on the younger population might be influenced by increased exposure for these workers. From very early stages in the pandemic, it was observed that the severity of the disease was greater in older individuals (21-23), and they were likely protected from exposure to some extent.”*

**Comment 12:**

6) Temporary employment agency activities seem like a rather diffuse category. Can you include some examples: e.g.: “For example, Temporary employment agency activities, including occupations such as X, Y and Z, recorded a rate 9 times ….”

**Reply:** We agree with your point of view, so we have included information in the text that better describes this category:

*“Regarding Temporary employment agency activities, several high risk occupations were identified for women, such as Agricultural labourers, Call centers operators, Fruit and vegetable preservers and sellers, whereas Transport and manufacturing labourers, Craft workers and Butchers, were high risk occupations for men.”*

**Comment 13:**

**General remarks:**

1) English should be written better and spelling errors must be checked. E.g. disability is written with one T, not two. Words like “objetive” instead of “objective”; and “previosly” instead of “previously”.

**Reply:**

Errors have been corrected.

**Reviewer #3:**

**Comment 1:**

**Introduction**
In the introduction, in order to make the problem understandable and to introduce readers to the presented COVID absenteeism problem in Spain, some international (European) comparisons are useful. The study sounds like a report in some places. In the introduction more motivations is useful, i.e. about the covid preliminary socio-economic consequences to Spanish society.

**Reply:**
We agree with your observations and have included information on the socio-economic consequences in Spain:

*“The most affected sectors in Europe (hotels, catering, transport and leisure) represent around 9% in the euro area, but their weight is higher in Spain (13%) or Italy (11%) (2). Studies carried out in Spain mention that household consumption decreased by 12.4%, while public spending increased by 3.8%. The investment sector dropped by 12.4%; construction and capital goods also dropped by 14% and 13%, respectively (3).”*

We updated the bibliography in order to include these papers.

**Comment 3:**

3) "...in quarantine and observation due to close contact with cases, and especially sensitive (vulnerable) workers..." What does "especially sensitive (vulnerable) workers" mean? please explain.

**Reply:** We have included information to show the type of population that was considered particularly vulnerable and the reasons why we have not analysed this group further.

*“Three situations were identified for workers protection: sick with COVID-19, in quarantine and observation due to close contact with cases, and especially sensitive (vulnerable) workers (cardiovascular disease, including hypertension, chronic lung disease, diabetes, chronic kidney failure, immunosuppression, cancer in the active treatment phase, severe chronic liver disease, morbid obesity (BMI>40), pregnancy and those over 60 years of age). The latter group (vulnerable workers) was not analysed to avoid bias in the study.”*

**Comment 4:**

4) "In previous studies, different..." In order to understand and evaluate the research gap that this paper fills, more details about these studies are useful.

**Reply:**

Following your observations and the comments of reviewer 2, we have included a paragraph to explain the newness of the article, as well as the absence of information that needs to be analysed to understand the real impact of the pandemicon sectors other than the health sector:

*“In previous studies, different papers have described the impact of COVID-19 in terms of TD in the healthcare and sociosanitary population (7-12). However, it is important to highlight that the beginning of the second wave in Spain and later in Europe had its origin in Agriculture seasonal workers (6). This shows the relevance of monitoring the work settings and the working and living conditions of workers to better understand the COVID-19 transmission mechanisms. Notwithstanding this, there are very few studies describing the magnitude of the impact on the non-healthcare working population (6, 13, 14). Consequently, our current knowledge of the COVID-19 impact in these workers is still incomplete.”*

**Methodology**

**Comment 5:**

1) "The ratio between TD due to quarantine and TD due to illness, defined as the ratio between TD due to quarantine and TD due to illness in the last 7 days, was calculated. For every economic sector of activity the median and interquartile range (IQR), representing the difference between the third quartile (Q3) and the first quartile (Q1), were calculated. This allowed comparing the dispersion among sectors. Population figures affiliated with social security, disaggregated at 1, 2, and 3 NACE digits, were used as denominators for rates calculations (15). To calculate rates by occupation, figures of employed individuals at 1, 2, and 3 ISCO digits were used, provided by the Economically Active Population Survey (16)."

In order to make the paper reader friendly (also for foreginers), these figures are useful. In addition, the equations for the mentioned ratios and statistical measures are necessary.

**Reply:**

We have included the formula of the intercuartile range ($IQR=Q3-Q1$) and the formulas for calculating the TD rates.

**Comment 6:**

2)"The following rates of COVID-19 temporary..." equations needed.

**Reply:**

We have included the equations according to your suggestion.

**Results**

**Comment 7:**

1) "Among the remaining cases, 74.4% (837,787) were due to quarantine from close contact with COVID-19 cases, and 25.6% (288,969) were due to illness. Of the TD processes, 54.6% (615,083) were observed in men (representing 54% of the studied
working population), and 45.4% (511,672) in women (46% of that working population). The TD rate reached values of 5,465 quarantines and 1,878 illnesses per 100,000 women, and 4,883 quarantines and 1,690 illnesses per 100,000 men."

In the introduction, the authors mention three groups to be analyzed in the study, two of which are presented here (" and especially sensitive (vulnerable) workers." are omitted).

**Reply:**

Vulnerable workers were omitted because of the nature of their condition, and not because of diagnosis or contact with a COVID-19 case. For this reason, they were not included in the analysis in order to avoid bias in our study. We added a phrase in the introduction in order to clarify this aspect:

*“Three situations were identified for workers protection: sick with COVID-19, in quarantine and observation due to close contact with cases, and vulnerable workers (cardiovascular disease, including hypertension, chronic lung disease, diabetes, chronic kidney failure, immunosuppression, cancer in the active treatment phase, severe chronic liver disease, morbid obesity (BMI>40), pregnancy and those over 60 years of age). The latter group (vulnerable workers) was not analysed to avoid bias in the study.”*

**Discussion**

**Comment 8:**

Please separate discussion from conclusions.

**Reply:**

In accordance with your suggestion, we have separated the conclusions from the discussion.

**Comment 9:**

2) Add some directions for future research

**Reply:**

In response to your suggestion, we have included some proposals for research that could be carried out as a result of this study:

“*It would be important to do some targeted research on the most affected groups in order to determine specifically what other factors might have influenced the increased incidence in some groups of non-health workers.”*

**Comment 10:**

3) "The higher impact on the younger population might be influenced by increased exposure, because from very early stages in the pandemic, it was observed that the disease was more severe in older individuals, and they were likely protected from exposure to some extent"

Are there any motivations in literature? Is this situation specific to Spanish society? Please explain.

**Reply:**

We have added three references to support this information, according to your suggestion:

"*The higher impact on the younger population might be influenced by increased exposure for these workers. From very early stages in the pandemic, it was observed that the severity of the disease was greater in older individuals (21-23), and they were likely protected from exposure to some extent."*

**Comment 11:**

These three paragraphs should be references in the literature.

4) "The results also show the uneven impact of COVID-19 by the economic activity of the Company or the occupation of the worker. The greatest impacts were observed in sectors such as *Activities of households as employers of domestic personnel, Temporary employment agency activities, Waste Collection, Activities of call centres, and Processing and preserving of meat and meat products,* essential sectors that have not interrupted their on-site work at any point in the evolution of the epidemic, either because telework is not possible (or more challenging), or
because the activity has been more intense due to the impact of the pandemic.(XX)

In this group of activities, it is worth noting the TD rate for domestic employees (NACE T and 97), which is unusually high, especially in women, likely due to an overestimation resulting from not including all workers in the sector in the denominator. It should be noted that affiliation data does not include special regimes, which may be numerous for this group. On the other hand, we have excluded and do not show the rates corresponding to Activities of extraterritorial organizations (NACE U) and Armed Forces Occupations (ISCO Q), as they present extremely small TD values, 99 and 39 processes, respectively, likely due to a notification bias.

Sectors such as Education or Food and beverage service activities show a lower impact. In the case of Education, during the second wave, TD increased from late August and early September, possibly indicating the massive screenings that many authorities carried out on teachers before the start of the school year. In the case of Food and beverage service activities, it should be considered that a large number of establishments were closed during both the first and second waves, reducing the professional exposure of this group to COVID-19 and the incidence of TD"(XX)

**Reply:** Done. We have included the corresponding references in the paragrahs and in the bibliography.

6. PLOS authors have the option to publish the peer review history of their article ([what does this mean?](https://journals.plos.org/plosone/s/editorial-and-peer-review-process#loc-peer-review-history)). If published, this will include your full peer review and any attached files.


If you choose “no”, your identity will remain anonymous but your review may still be made public.

**Do you want your identity to be public for this peer review?** For information about this choice, including consent withdrawal, please see our [Privacy Policy](https://www.plos.org/privacy-policy).

Reviewer #1: **Yes:** Dr. med. Falk Liebers, MSc.

Reviewer #2: **Yes:** Fredrik Methi

Reviewer #3: No

[NOTE: If reviewer comments were submitted as an attachment file, they will be attached to this email and accessible via the submission site. Please log into your account, locate the manuscript record, and check for the action link "View Attachments". If this link does not appear, there are no attachment files.]

While revising your submission, please upload your figure files to the Preflight Analysis and Conversion Engine (PACE) digital diagnostic tool, <https://pacev2.apexcovantage.com/>. PACE helps ensure that figures meet PLOS requirements. To use PACE, you must first register as a user. Registration is free. Then, login and navigate to the UPLOAD tab, where you will find detailed instructions on how to use the tool. If you encounter any issues or have any questions when using PACE, please email PLOS at [figures@plos.org](mailto:figures@plos.org). Please note that Supporting Information files do not need this step.
